# Are We There Yet? Exploring the Impact of Translating Cognitive Tests for Dementia Using Mobile Technology in an Aging Population

**DOI:** 10.3389/fnagi.2016.00021

**Published:** 2016-03-17

**Authors:** Kai Ruggeri, Áine Maguire, Jack L. Andrews, Eric Martin, Shantanu Menon

**Affiliations:** ^1^Policy Research Group, Department of Psychology, University of CambridgeCambridgeshire, UK; ^2^Engineering Design Centre, Department of Engineering, University of CambridgeCambridgeshire, UK; ^3^Department of Psychology, University of CambridgeCambridgeshire, UK; ^4^Department of Engineering, University of CambridgeCambridgeshire, UK

**Keywords:** dementia, screening, technology, mHealth, policy, cognitive function, computerized testing

## Abstract

This study examines implications of the expanded use of mobile platforms in testing cognitive function, and generates evidence on the impact utilizing mobile platforms for dementia screen. The Saint Louis University Mental State examination (SLUMS) was ported onto a computerized mobile application named the Cambridge University Pen to Digital Equivalence assessment (CUPDE). CUPDE was piloted and compared to the traditional pen and paper version, with a common comparator test for both groups. Sixty healthy participants (aged 50–79) completed both measurements. Differences were tested between overall outcomes, individual items, and relationship with the comparator. Significant differences in the overall scores between the two testing versions as well as within individual items were observed. Even when groups were matched by cognitive function and age, scores on SLUMS original version (*M* = 19.75, *SD* = 3) were significantly higher than those on CUPDE (*M* = 15.88, *SD* = 3.5), *t*_(15)_ = 3.02, *p* < 0.01. Mobile platforms require the development of new normative standards, even when items can be directly translated. Furthermore, these must fit aging populations with significant variance in familiarity with mobile technology. Greater understanding of the interplay and related mechanisms between auditory and visual systems, which are not well understood yet in the context of mobile technologies, is mandatory.

## Introduction

The World Alzheimer's Report (2015) estimates that there are 9.9 million new cases of dementia each year, and that the global annual cost of the disease will reach US $818 billion this year (Prince et al., 2015). Such estimates are routinely used by policy makers for the provision of services and supports for those affected, and have spurred on increasing research into potential mechanisms to prevent and inhibit disease progression, as well as interventions to reduce the consequences of the disease. Given these arguments, there are continued discussions calling for better detection concurrently with concerns regarding approaches to population screening. Therefore, it is important to have clear evidence on best practice if such approaches continue and expand.

Early detection of conditions such as Mild Cognitive Impairment (MCI) that may precede the onset of dementia could potentially reduce this global burden, by providing timely access to appropriate treatments and interventions, as well as facilitating social support and greater understanding of symptom progression for families and persons diagnosed (Ashford et al., 2007). However, MCI, although a potential predictor for the development dementia, is not actually an early stage of dementia (Richard and Brayne, 2014), and its early signs have little sensitivity or predictive utility for later diagnosis of dementia (Roberts et al., 2014). Rather, recent research has highlighted the potential pitfalls of widespread screening for MCI and dementia which include inappropriate treatment with pharmacological agents that have shown limited evidence for disease modification, adverse social outcomes such as stigmatization, misallocation of services and supports from those with marked dementia, as well as the financial cost of administering assessments within the primary care setting (Couteur et al., 2013; Fox et al., 2013).

At present the true benefits of diagnosing MCI in the general population relative to its harms have not been fully analyzed, and there is insufficient evidence to support either argument for or against its use in screening for dementia (Fox et al., 2013; Richard and Brayne, 2014). As such although the utility of diagnosing MCI should not be underestimated, any approach to screening within general population should be taken with extreme caution. The need for cost-effective, accurate tests to detect cognitive function and associated cognitive decline in an aging population is greater than ever (Cullen et al., 2007; Handels et al., 2015).

Computerized neuropsychological assessment devices (CNADs) have been increasingly used in attempts to reduce the costs of large-scale screening for age-related cognitive decline whilst reaching a wider proportion of the population at risk (Bauer et al., 2012). These devices offer a number of potential benefits relative to tradition pen and papers assessments for illness screening, including the ability to alter the tests to accommodate for different languages, the reduced need for trained professionals to administer the tests, the ability to implement immediate performance adjustment, the automatic exportation of data, and the ability to measure performance on time sensitive tasks. For example, the Automatic Cognitive Assessment Delivery (ACAD) is a reliable and validated home-based online assessment of memory and attention that is free from learning effects and is suitable for use in an older population due to it relatively simple format and clear instructions (Di Rosa et al., 2014). However, such advantages of CNADs may also be at the cost of other measures gained through traditional administration methods, such as the reduced ability to collect qualitative data and the lack of speech quality measures (Wild et al., 2008; Bauer et al., 2012).

In recent years, there has been an influx of online and computer based screening tools for dementia and other condition. Although, some have been specifically developed and validated against a plenitude of established screening measures (e.g., ACAD), others have simply been translated from traditional pen and paper format onto a digital platform without transparent validation methods (e.g., Addenbrooke's Cognitive Examination Mobile). The latter is concerning given the prevailing outcry that existing paper-based tests should not be translated to computerized formats on face validity alone (Schlegel and Gilliland, 2007; Wild et al., 2008; Zygouris and Tsolaki, 2015). They must meet the same standards of development and use as paper-based tests administered by an examiner, and it cannot be assumed that the same normative data collected for traditional style tests can be directly applied to computer based versions of the same test (Bauer et al., 2012). Without established psychometric quality, standardization, and administration advice these devices increase the risk of poor clinical decision-making (Gates and Kochan, 2015).

Assessment methods can potentially change the cognitive functions being tested, there is a risk of confounding results between traditional testing and computerized adaptations. Features of user interface (such as the way information is conveyed, the way stimuli are presented, and the way responses are inputted) can hinder user ability answer the same question consistently on different platforms (Errey et al., 2006). We document evidence emphasizing the need for better validation and psychometric properties of neuropsychological tests aimed at elderly populations, which are traditional pen and paper tests translated to digital platforms.

The Saint Louis University Mental State examination (SLUMS) (Tariq et al., 2006), a pen and paper test for MCI and dementia, is used as a proxy to empirically demonstrate some of the theorized issues that affect the translation of traditional tests to computerized platforms. This study involves healthy older individuals that would ordinarily be used to provide normative data for such tests. The translation of SLUMS to a computerized mobile application is named the Cambridge University Pen to Digital Equivalence assessment (CUPDE). This study aims to demonstrate potential normative differences between a traditional test of cognitive function and its translation to a computerized, mobile application. It emphasizes the limitations of using such tools to detect clinically relevant symptoms in an older population, and reiterates concerns raised by clinicians, policy makers, and other researchers regarding the use of widespread screening for MCI within the general population. The aim is to understand the differences and challenges while presenting clear guidance on next steps for both further study and, as is already likely, application.

## Materials and methods

### Participants

A total of sixty participants between 50 and 79 years of age (*M* = 61.37, *SD* = 6.44) completed this study. All participants were volunteers and were recruited via convenience sampling from locations in Surrey and Cambridgeshire. Inclusion criteria for this study were participants who were native English speakers, had normal or corrected visions, and living independently. Exclusion criteria included the presence of neurological disease or disorder, and reported history or presence of memory complaints, psychiatric disorder or neurological disease. This study was approved by academic and clinical ethical boards at the University of Cambridge. Informed consent was provided by all participants.

### Procedure

Participants were randomly assigned to one of two conditions. The first completed SLUMS (*N* = 30), administered by a researcher, the second CUPDE (*N* = 30), presented via an iPad application. For validation and comparative purposes, both groups were also required to complete the Self-Administered Gerocognitive Exam (Scharre et al., 2010) after taking either SLUMS or CUPDE. For participants completing the SLUMS assessment, researchers filled in participant details and subsequently followed the protocol advised by the developers of SLUMS. For participants completing the CUPDE assessment, participants filled in details and assessments individually. Participants followed onscreen instructions adapted from the SLUMS protocol. After testing, all CUPDE participants completed a usability questionnaire to capture any potential confounds. Testing took no longer than 45 minutes for each condition.

### Measures

#### Saint Louis University mental state examination (SLUMS)

SLUMS is an eleven-item instrument that tests a variety of cognitive abilities, with a UK-specific version available in English. It is a reliable screening tool for MCI and dementia that has demonstrated validity, and has fewer ceiling effects than the widely-used Mini Mental State Exam (MMSE) (Feliciano et al., 2013). Permission to port SLUMS to a computerized mobile format was acquired from the SLUMS authors (Tariq et al., 2006).

#### Cambridge University pen to digital equivalence assessment (CUPDE)

CUPDE, the digital format of SLUMS, was built using JavaScript, HTML and CSS code with all questions from SLUMS except one being directly ported (the question wording and required response were the same across both platforms, although the delivery mode may have differed). Testing was conducted on a web browser (Safari) on a mobile, handheld tablet (iPad). The test was transformed in a way that aimed to best represent a direct conversion of the traditional SLUMS assessment, although in this phase of development a number of discrepancies resulted in terms of sensory and motor input and output functions. The SLUMS assessment is delivered orally with participants responding verbally to nine items (Qs.1–8 and Q.11), with two drawing items completed manually (Q.9 and Q.10). The items in CUPDE were presented via text, where the participant was required to read the items before responding (Qs.1–3, Q.6, Q.9, Q.10), audio files (Q.4, Q.7, Q.8) or both (Q.5, Q.11) and participants were required to respond to all items by selecting buttons or using a non-predictive onscreen keyboard.

One item (Q.6) could not be adequately ported due to technical limitations related to testing on an iPad. This requires participants to list as many animals as possible in 1 min and is designed as a measure of semantic fluency, a measure which is thought to be a sensitive indicator of early stage dementia (Zhao et al., 2013). Due to technical limitations that would have affected score accuracy, a word classification task was used in this place in the CUPDE version. Words randomly selected from a master list of nouns were displayed individually, requiring participants to use buttons to record whether the words were animals or non-animals. A 35-second limit was placed on the tablet version, as opposed to 60 seconds in the traditional version. All participants saw the same list in the same order.

#### Standard neuropsychological test: Self-administered gerocognitive exam

In order to compare the concurrent validity of CUPDE and SLUMS, as well as the differences between the two formats, the Self-Administered Gerocognitive Exam (SAGE) (Scharre et al., 2010) was used. SAGE is a traditional self-administered pen and paper test, which aims to identify MCI from any cause, as well as early onset dementia. It has greater sensitivity than several other tests of cognitive decline such as the MMSE (Scharre et al., 2010), making it easier to pick up very minor impairments of cognition.

### Dependent variables

The SLUMS examination was scored using the standard guidelines with education protocols applied to final results, with a maximum score of 30. The same scoring was applied for CUPDE, though the modified item used standardized results from the group, with item scores categorized into quartiles in order to best match the scoring system of SLUMS.

## Results

Data were analyzed to present an overall description of the trial as well as for direct comparison. In order to compare overall results between conditions (Table 1), naïve difference tests were used on all data. To then control for potential group differences, paired tests were run on participants from the two groups after being matched for SAGE scores and age. Normality tests (Shapiro–Wilk and Kolmogorov–Smirnov tests) indicated that the SAGE scores were not normally distributed, as such non-parametric analyses were performed when using SAGE scores. This was anticipated given it was a healthy sample.

**Table 1:** **Results for cognitive tests used**.

	**Mean**
Condition A: SLUMS^a^	22.53 (3.32)
Condition B: CUPDE^a^	16.8 (4.13)
Condition A: SAGE^a^	18.83 (2.25)
Condition B: SAGE^a^	17.87 (3.19)
	**ρ**
SAGE and SLUMS^a^	0.54^**^
SAGE and CUPDE^a^	0.44^*^
	***t(df)***
SLUMS and CUPDE^b^	5.93 (58)^***^

a *n = 30*.

b *n = 60*.

* p < 0.05;

** p < 0.005;

*** *p < 0.001*.

### Comparison of experimental groups

There was no significant difference in SAGE scores between participants in the SLUMS (*Md* = 18.5, *n* = 30) and CUPDE groups, (*Md* = 19, *n* = 30); *U* = 403*, z* = –0.702*, p* = 0.48, *r* = 0.09. There was a strong, positive correlation between SLUMS and SAGE scores; *r*_*s*_ = 0.54, *n* = 30, *p* < 0.005. There was a moderate correlation between CUPDE and SAGE scores; *r*_*s*_ = 0.44, *p* < 0.05, *n* = 30.

### Differences between SLUMS and CUPDE

Naïve independent *t*-tests for total score showed a significant difference between SLUMS (*M* = 22.53, *SD* = 3.32) and CUPDE (*M* = 16.8, *SD* = 4.13); *t*_(58)_ = 5.93, *p* < 0.001. The magnitude of the differences in the means (mean difference = 5.73, 95% *CI* = 3.8–7.67) was large (eta squared = 0.38).

### Itemized comparisons between SLUMS and CUPDE

Table 2 presents differences in items scores between SLUMS and Question 1 was answered correctly by all participants. Significant differences were seen between SLUMS and CUPDE on question 3 (County), χ^2^(1) = 27.778, *p* < 0.05, question 11a (Story: Work), χ^2^(1) = 25.452, *p* < 0.05, question 11b (Story: Back to Work), χ^2^(1) = 13.611, *p* < 0.05, and questions 11d (Story: Country) χ^2^(1) = 12.273, *p* < 0.05. Question 6 was not computed, as the criterion for expected cell frequencies was not met. Using the standardized scoring approach and categorizing into quartiles to match the scoring system of SLUMS, 20% (6) of the CUPDE participants achieved the highest score of 3, whilst 93.3% (28) of participants in the SLUMS condition achieved a score of 3.

**Table 2:** **Itemised comparisons between SLUMS and CUPDE^a^**.

**Question**	***X*^2^**	***df***	***p***
1. Day of Week^b^	–	–	–
2. Year	1.017	1	0.313
3. County	27.778^**^	1	0.001
5. Calculation (Spent)	0.268	1	0.605
5. Calculation (Change)	0.069	1	0.793
6. Animals^c^	–	–	–
7. Objects^d^	7.938	5	0.160
8. Back digit	2.381	2	0.304
9. Clock Hours	3.360	1	0.067
9. Clock Time	0.268	1	0.605
10. Shape (Triangle)	2.308	1	0.129
10. Shape (Largest)	0.001	1	1.0
11a. Story (Name)	2.443	1	0.118
11b. Story (Work)	35.623^**^	1	0.001
11c. Story (Back to work)	6.696^*^	1	0.010
11d. Story (Country)	12.273	1	0.001

a *n = 60 for all items, with 30 in each group*.

b *All participants answered item one correctly in both conditions*.

c *Did not meet criteria for minimum expected cell frequency*.

d *Accounts for question 4 score as well*.

* p < 0.05;

** *p < 0.01*.

### Matched case analysis

**Figure 1:**
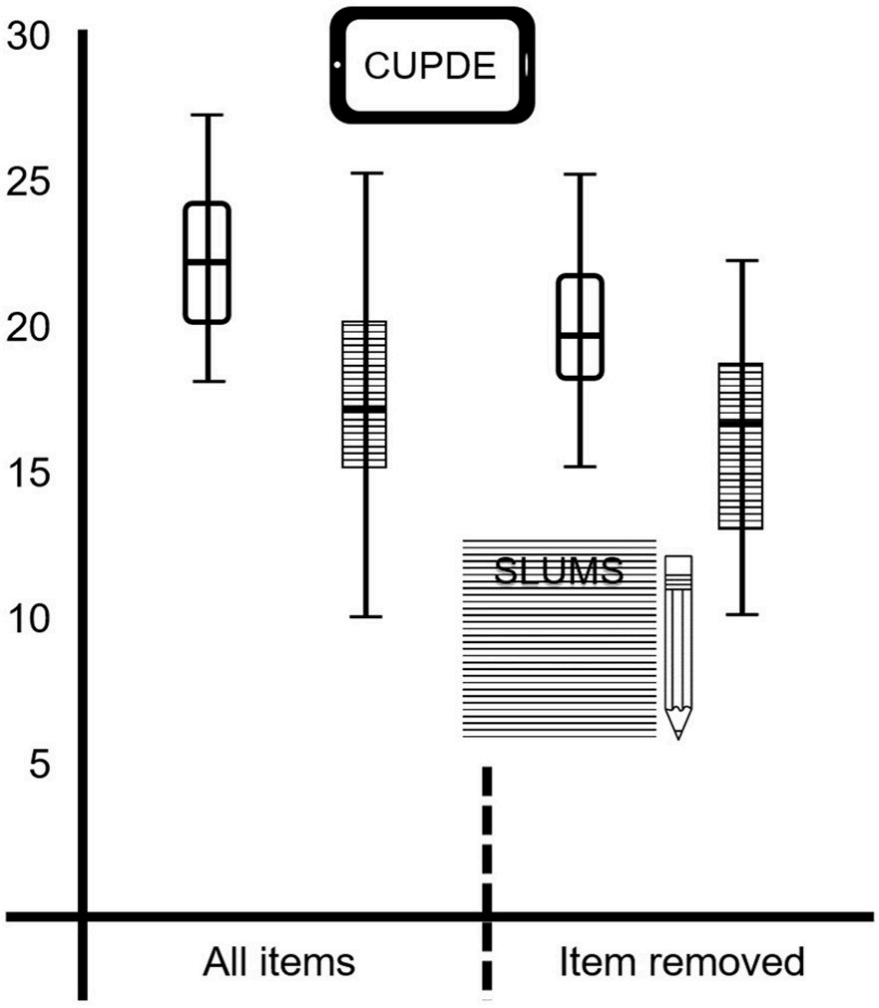
**Distributions of matched scores for SLUMS and CUPDE for total score and total without the modified item**.

When matched for cognitive function on SAGE scores, total score on SLUMS (*M* = 22.53, *SD* = 2.88) was significantly greater than on CUPDE (*M* = 17.29, *SD* = 4.01); *t*_(16)_ = 4.10, *p* < 0.005, 95% CI = 2.53–7.94. There was considerable overlap in the distributions for the measures (Figure 1), though scores on CUPDE were more varied. Analysis excluding question six to avoid differences caused by artifact showed a similar pattern in the results, with SLUMS scores (*M* = 19.75, *SD* = 3) significantly higher than CUPDE (*M* = 15.88, *SD* = 3.5); *t*_(15)_ = 3.02, *p* < 0.01; 95% CI = 1.14–6.61.

## Discussion

Using a sample of healthy older adults this study was intended to understand potential normative differences between a traditional test of cognitive function and its translation to a computerized, mobile application. Even in a relatively small sample, results on cognitive function tests differed significantly when translated from a pen and paper format to a computerized mobile tablet format. The difference could not be attributed to any underlying cognitive differences between groups and further analyses demonstrated a greater correlation between the standard neuropsychological test scores and SLUMS, the traditional version, than when the standard neuropsychological test scores was compared with CUPDE, the tablet version. Combined, these results suggest that the two testing formats deviate in their ability to predict the same mental constructs, even when accounting for modifications in the test. This is particularly noteworthy given that participants were highly similar on the standard neuropsychological test.

Question 6 from SLUMS assess declarative memory, specifically semantic memory processes as opposed episodic memory, and is used as measure of semantic fluency, which are considered sensitive indicators of early stage dementia (Destrieux et al., 2012). Due to an inability to employ accurate voice recognition software, the task was manipulated into a word classification test. Participants were asked if a particular word presented to them is an animal or a non-animal. The time restriction of 35 s appeared to be a very limiting space for the majority of participants to complete the new test. The restrictive time limit emphasizes participant reaction time, a factor that is absent from the paper version of this question. Thus, it is more likely that the item assessed participants' speed of information processing or level of sustained attention (McVay and Kane, 2012) rather than semantic fluency as was intended in the traditional format (Shao et al., 2014). The task no longer places a large emphasis on retrieval in the same way the category test of free recall does, and the item fails to demonstrate the same level of accuracy with regards to which cognitive processes the original item measures (Shao et al., 2014). Results on this item are more likely to reflect a combination of semantic visual word recognition abilities (Chee et al., 1999), grapheme to phoneme conversion abilities, motor inhibition or abilities, dyslexic profiles, and associated linguistic abilities.

The use of word classification over semantic fluency highlights an important limitation in this study in that matching performance is not sufficient nor appropriate in comparing cognitive test results. However, this is mentioned specifically to draw attention to the concerns raised about direct translation: considerable problems are likely to arise when even minor modifications have to be made when translating a traditional test into mobile form, though it is unlikely that all such concerns can be addressed in such a way that two versions can be considered as entirely equivalent. This flaw can be exacerbated when programming a test requires a large number of decisions in how to adapt a single question such that the question itself changes, not only how the answer is provided. In this context, the role of semantic fluency had been long established and variations in score were well-mapped within the SLUMS test. The shift to word classification, while similar in many respects, does not offer assumed parallels (either direct equivalence or a weighted algorithm of some kind).

More detailed difference testing was conducted on the results of the remaining items between the SLUMS and CUPDE conditions. The most significant differences were found on questions 3 and 11. Question 3 required participants to state the *county* that they were currently in. A large majority of participants (*n* = 22) in the tablet condition (CUPDE) wrongly interpreted this question as *country* and subsequently reported incorrect answers. This is potentially explained by the method of input. In the SLUMS condition, the researcher explicitly asks the question to the participant, who has the opportunity to request clarification. In contrast, the tablet device relies on the user accurately reading the word. Earlier research has suggested that individuals tend to read less accurately and comprehensively on screens than on paper (Dillon, 1992), which adds another layer of concern regarding the expanded use of self-administered mobile cognitive tests, if not appropriately accounted for.

It has been previously reported that perceptual expectancies may exist during reading tasks, where subjects read what they expect to see as opposed to the text that actually appears (Galitz, 2007). It is also possible that potential computer anxiety, which is frequently observed in elderly populations (Czaja et al., 2006), may have increased stress levels and subsequently reduced attention to the task at hand, eliciting such misinterpretations (Grady, 2012). Thus, when developing any computer or mobile based dementia assessment or screening tools it's suitability for use in today's aging population with varying degrees of computer and mobile technology experience must be considered.

Future translation studies should look to report results across a broader selection of participants, taking into account socioeconomic background and prior experience of technology whilst controlling for constructs such as motivation, personality and gender. For instance, while it was intentional to test only healthy individuals, it is possible that comparisons would look entirely different if expanded to those with known cognitive function deficits. This is particularly important when considering that the results of such individuals on the tablet version may be considerably worse and perhaps present skew not present with a pen/paper test. Whether this is a useful method for distinguishing health from decline is not possible to address within the context of this study. However, it does offer tangible insights into the likely influence of individual differences on many levels into performance on adapted versions of mobile tests. Prior experience with relevant technology will also provide significant insight when it comes to tasks such as typing on a mobile platform, which may be seen as a standard skill for digital natives, but cannot be assumed as equivalent for current older populations. Additionally, rather than using one common measure as a baseline assessment of cognitive function, subsequent work should look at comparing participant baseline functioning across a variety of assessment measures.

Equivalence testing has indicated that when a pen and paper test of cognitive function is translated to a computerized mobile format, the result is effectively a completely new test. To reflect the differences in cognitive functions being tested, a bespoke scoring system must be designed for a translated mobile-based test. A computerized test places a different set of cognitive demands on an elderly user than a traditional paper test. The scoring system of the paper test is designed to evaluate the performance of specific cognitive functions in the user. Testing the identical question on a computerized platform changes the cognitive functions being targeted by the identical question presented digitally, due to the additional cognitive loads imposed by the visual design, user interface, input/output modality etc. Therefore, CNADs built from translated paper tests cannot simply apply the same scoring system as the original paper test without further consideration. It is also an opportunity to elicit perhaps more useful data, such as timing for completing each item, hand tremors, visual acuity, and other motor skills (or proxies thereof). Such approaches to testing are already prominent within neuropsychology. Furthermore, clinical neuroscience may offer significant insight pertaining to the differing activation of brain regions that may be impacted, which is also relevant for approaches to scoring.

Developing a set of normative standards for a tablet device is likely to be more difficult than the equivalent paper test due to the additional layer of complexity added by the user interface and the variation that can exist in the elderly population's comfort, familiarity and trust in technology. If a translated test is to be used for clinical assessment, then it is of great policy relevance for new normative data to be collected. We argue that whilst new normative data might perhaps generate the ability for a test to show differences between typical and atypical scores, such translations may in fact be testing dissimilar cognitive constructs. This is demonstrated in the case of SLUMS, with SAGE correlating well with SLUMS but less so with the translated version, CUPDE. Tests that have been standardized solely within a computerized or tablet version have already addressed this concern, so it is primarily focused toward translations.

Inclusive and intrinsically motivating design will be exceptionally important if CNADs become part of worldwide mental screening programs and start to reach a stage where they can fulfill the current promise of fast, efficient, widespread, easily accessible, and highly reliable cognitive screening for the population (Wollner et al., 2012). As these tests expand in use, user interfaces must also be adapted to fit a diverse population. The operation of this interface must take up as little user cognitive faculties as possible to minimize interference with the parallel demands being placed on the user by the actual neuropsychological test, all while maintaining equivalence across platforms. Further neuropsychological and neuroscientific evidence relating to the cognitive and physiological differences elicited by such translations would provide a positive impact on clinical practice.

We call for a greater body of research into the effects of translating pen and paper tests to computerized versions, specifically on establishing norms and thresholds for early detection when using mobile devices. Clinical neuropsychological and experimental research should strive to further map the cognitive processes that are best tested by pen and paper vs. computer based assessments. If it is reported which cognitive processes are best studied through traditional or computerized tests, then subsequent neuropsychological diagnoses will undoubtedly benefit from such increases in sensitivity and specificity. Additionally, those concerns raised about the appropriateness of screening programs for cognitive function should be considered within these steps.

Additional steps for such uses may include considering how good mental health could be promoted through the use of mobile technology, as such expansion of access to individuals presents opportunities beyond simply creating new products to confirm illness, but could offer more to encouraging healthy behavior across a population. As better evidence comes available from the mobile technology and mental health movement, or mH^2^ (Farrington et al., 2014), such applications create great potential for impact beyond detection systems.

With sufficient information covering these domains, policymakers, clinical practitioners, and health service providers will be better placed when it comes to decision-making on appropriate tests for understanding and addressing mental health in an aging population, if use of such tests continues. Specifically, policymakers should require evidence not only on testing elements involved in specific instruments, but also how scoring has been developed specifically considering the medium used, especially if it involves very modern technology within a population not considered as digital natives. This will ensure resources are used effectively and only on tools that have been validated on all relevant levels. Only at this point should there be a consideration to apply on a large scale, if it is to happen at all. This presents an opportunity for neuroscientists and cognitive psychologists to play a significant role in effective, stratified and evidence-based mental health policy.

## Ethics statement

Due to multiple department involvement, ethical review was provided by both the Engineering Design Centre and the Department of Psychology at the University of Cambridge. The study was covered by the University of Cambridge Public Liability insurance. All participants provided consent at the beginning of the test.

## Author contributions

KR directed the project, development, procedure, ethical review, writing, and submission. JA participated in development, statistical analysis, and writing. EM participated in development and writing. SM participated in development, data collection, and writing. ÁM contributed to the development, procedure, data collection, statistical analysis, writing, and submission. All authors agree to be accountable for all aspects of the work and gave final approval for this version to be published.

## Funding

Support for the dissemination of this work was provided by the UK Economic and Social Research Council ES/LO14629/1.

### Conflict of interest statement

The authors declare that the research was conducted in the absence of any commercial or financial relationships that could be construed as a potential conflict of interest.
